# Interaction Mechanism of Composite Propellant Components under Heating Conditions

**DOI:** 10.3390/polym15112485

**Published:** 2023-05-28

**Authors:** Jiahao Liang, Jianxin Nie, Haijun Zhang, Xueyong Guo, Shi Yan, Ming Han

**Affiliations:** 1 State Key Laboratory of Explosion Science and Technology, Beijing Institute of Technology, Beijing 100081, China; 2 Xi’an Modern Control Technology Research Institute, Xi’an 710065, China; 3 The Eighth Military Representative Office of Air Force Equipment Ministry, Beijing 100843, China

**Keywords:** composite propellant, thermal damage treatment, thermal weight loss, interaction, combustion characteristics

## Abstract

To examine the interactions between two binder systems—hydroxyl-terminated polybutadiene (HTPB) and hydroxyl-terminated block copolyether prepolymer (HTPE)—as well as between these binders and ammonium perchlorate (AP) at various temperatures for their susceptibility to varying degrees of thermal damage treatment, the thermal characteristics and combustion interactions of the HTPB and HTPE binder systems, HTPB/AP and HTPE/AP mixtures, and HTPB/AP/Al and HTPE/AP/Al propellants were studied. The results showed that the first and second weight loss decomposition peak temperatures of the HTPB binder were, respectively, 85.34 and 55.74 °C higher than the HTPE binder. The HTPE binder decomposed more easily than the HTPB binder. The microstructure showed that the HTPB binder became brittle and cracked when heated, while the HTPE binder liquefied when heated. The combustion characteristic index, *S*, and the difference between calculated and experimental mass damage, ΔW, indicated that the components interacted. The original *S* index of the HTPB/AP mixture was 3.34 × 10^−8^; *S* first decreased and then increased to 4.24 × 10^−8^ with the sampling temperature. Its combustion was initially mild, then intensified. The original *S* index of the HTPE/AP mixture was 3.78 × 10^−8^; *S* increased and then decreased to 2.78 × 10^−8^ with the increasing sampling temperature. Its combustion was initially rapid, then slowed. Under high-temperature conditions, the HTPB/AP/Al propellants combusted more intensely than the HTPE/AP/Al propellants, and its components interacted more strongly. A heated HTPE/AP mixture acted as a barrier, reducing the responsiveness of solid propellants.

## 1. Introduction

Hydroxyl-terminated polybutadiene (HTPB) propellant is currently the most important type of composite propellant and has been applied in various rocket motor models in China and abroad. HTPB not only improves the specific impulse of the propellant but also has a widely adjustable range of burning rates, good mechanical properties, a simple manufacturing process, and abundant raw materials [1,2,3,4,5,6]. Thus, it is one of the mainstream composite propellants in use. However, a new type of insensitive solid propellant has been developed that uses hydroxyl-terminated block copolyether prepolymer (HTPE) as a binder. It has good desensitisation performance, energy characteristics, and application performance [7]. Owing to its significant insensitivity under hazardous conditions such as slow heating, HTPE propellant is intended to replace HTPB propellant [8,9,10,11,12]. Based on their performance, HTPB and HTPE propellants can be applied in a wide range of environments. However, with the increasing complexity of the service environment, the possibility of accidental reaction and the degree of harm of the motor in the process of use is also increasing. Therefore, it is an important development direction to improve the survivability of solid rocket motors in a complex environment in the future.

A composite solid propellant, which has certain mechanical properties, is manufactured by mixing and curing oxidants (such as ammonium perchlorate (AP) and ammonium nitrate), combustion agents (such as Al powder), and polymer binders. Unexpected thermal decomposition and the energy release of propellants can occur in stimulating environments subjected to heating conditions. A typical example is an external fire causing a warehouse to warm up slowly; the heating process can lead to the thermal decomposition of the propellant. The thermal decomposition behaviour of propellants, especially the interaction between components, significantly affects their combustion characteristics after ignition. The thermal decomposition process is defined as the initial stage of ignition and combustion [13], and the decomposition characteristics of propellants have a profound effect on their combustion characteristics [14]. Therefore, the thermal analysis and cocombustion of energetic materials, such as solid propellants, is crucial not only to understand the thermal decomposition behaviour of propellants but also to conduct an in-depth evaluation of the effect of propellant exothermic decomposition on potential hazards during heating processes [15,16].

The thermal decomposition and combustion characteristics of propellants are closely related to the interactions between other components of the propellant [17]. Extensive research has been conducted on the thermal decomposition properties of AP [18,19], HTPB [20,21,22,23], HTPE [24], HTPB/AP propellant [25,26], and HTPE/AP propellant [16,27,28], laying an important foundation for understanding the thermal decomposition, ignition, and combustion characteristics of HTPB and HTPE propellants. However, similar research has not been conducted on the interaction between the propellant components during heating. During the heating process, various degrees of thermal decomposition of the propellant components occur, and their interactions are variable. Therefore, understanding the interactions between the components during the heating process is crucial for understanding the thermal decomposition behaviour of propellants and the effect of exothermic decomposition on potential hazards.

In this study, the interactions and cocombustion between two binder systems (HTPB and HTPE) at various temperatures were studied. The interactions and cocombustion between the incremental components of the propellants were evaluated by using thermogravimetric–Fourier transform infrared–mass spectrometry (TG–FTIR–MS) and other testing methods. In addition, the interactions between the binders and AP were analysed, which can provide a theoretical basis for further understanding and research on the thermal safety of propellants under heating conditions.

## 2. Materials and Methods

### 2.1. Materials

To study the interaction and cocombustion between propellants and their components via thermal analysis characteristics, the following incremental component formulations were designed: HTPB binder, HTPB/AP mixture, HTPB/AP/Al propellant, HTPE binder, HTPE/AP mixture, and HTPE/AP/Al propellant. The mass ratio of the binder to AP particles in the mixture and propellant was 18:82, and the composition and content of their respective formulations are listed in Table 1. The samples used in this experiment were developed and prepared by the Beijing Institute of Technology.

### 2.2. Equipment and Conditions Methods

To analyse the interaction and cocombustion between the components of the propellant, an experimental device for slow-heating propellant tables [29] was used to heat the samples (Table 1) at a rate of 0.2 °C/min. One sample was removed when the temperature listed in Table 2 was reached. The heated samples were analysed by using a simultaneous thermal analyser infrared–mass spectrometer (Netzsch—STA449F3, FTIR Nicolet iS20, Netzsch—QMS 403, Beijing, China, accessed from www.eceshi.com, accessed on 14 February 2023.). Approximately 3.0 mg of the sample was heated from an initial temperature of 45 °C to 800 °C at a rate of 10 K/min. The purge gas was high-purity argon with a gas flow rate of 240 mL/min. The testing range of the mass spectrometer was 0–300 *m*/*z*. Scanning electron microscopy (SEM; Hitachi, S-4800, Beijing, China, accessed from www.eceshi.com, accessed on 15 May 2023.) was used to visually analyse the micromorphology of the components of the propellant.

## 3. Results and Discussion

### 3.1. Thermal Decomposition of a Single-Component Propellant

#### 3.1.1. Thermal Decomposition of the HTPB Binder at Various Sampling Temperatures

**Figure 1:**
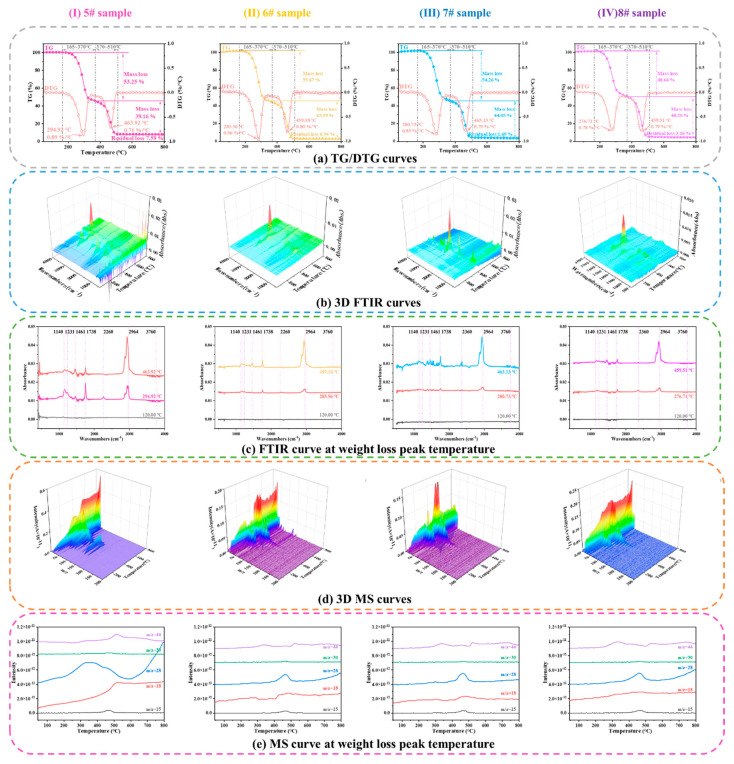
TG−FTIR−MS curves of the HTPB binder films heated to various temperatures.

Figure 1 shows the TG–MS–FTIR curves of the HTPB binder films heated to various temperatures (original samples and samples with sampling temperatures of 160 ℃, 180 ℃, and 220 ℃, respectively). The TG/DTG curve (differential thermogravimetry (DTG), a curve that differentiates each point on a TG curve with respect to temperature coordinates to the first degree) in Figure 1a shows that the thermal weight loss process of the film was completed in two stages.

For sample 5# HTPB binder, the first stage of weight loss occurs between 165 and 370 °C, with a maximum peak temperature at 294.92 °C for weight loss decomposition and a maximum weight loss rate of 0.89%/°C. The second stage occurs between 370 and 510 °C, with a maximum peak temperature at 463.92 °C for weight loss decomposition and a maximum weight loss rate of 0.71%/°C. The first stage occurs mainly because of the decomposition and volatilisation of DOA and TDI in the film when heated, and the second stage occurs mainly because of the chain-breaking decomposition and volatilisation of the HTPB polymer [30].

For sample 6#, the first stage of weight loss occurs between 165 and 370 °C, with a weight loss of 55.67%. The maximum peak temperature for weight loss decomposition is 285.56 °C, and the maximum weight loss rate is 0.96%/°C. The second stage occurs between 370 and 510 °C. During this stage, the weight loss is 43.39%, the maximum peak temperature of weight loss decomposition is 459.18 °C, and the maximum weight loss rate is 0.80%/°C.

For sample 7#, the first stage of weight loss occurs between 165 and 370 °C, with a weight loss of 54.26%. The maximum peak temperature for weight loss decomposition is 280.73 °C, and the maximum weight loss rate is 0.85%/°C. The second stage occurs between 370 and 510 °C, with a weight loss of 44.05%, maximum peak temperature of weight loss decomposition of 463.13 °C, and maximum weight loss rate of 0.79%/°C.

For sample 8#, the first stage of weight loss occurs between 165 and 370 °C, with a weight loss of 48.64%. The maximum peak temperature for weight loss decomposition is 276.71 °C, and the maximum weight loss rate is 0.78%/°C. The second stage occurs between 370 and 510 °C. During this stage, the weight loss is 48.20%, the maximum peak temperature of weight loss decomposition is 459.51 °C, and the maximum weight loss rate is 0.79%/°C. These results indicate that as the sampling temperature increases, the peak temperature of the first stage of weight loss of the HTPB films and their weight loss rate slightly decrease, but almost no effect is observed in the second stage of weight loss.

The weight loss peak temperatures of the HTPB binder in the FTIR curve reveal the corresponding groups of each characteristic absorption peak as follows: 2874–2964 cm^−1^ for C-H (2964 cm^−1^ for the asymmetric stretching vibration peak of C-H on CH_3_ and 2874 cm^−1^ for the symmetric stretching vibration peak of C-H on CH_3_); 2260 cm^−1^ for N_2_O; 1738 cm^−1^ for the stretching vibration peak of C=O; 1461 cm^−1^ for the in-plane bending vibration peak of C-H on CH_2_; 1231 cm^−1^ for the amide band III (this peak also represents the stretching vibration peak of C-N, which is a strong characteristic of polyurethane when it exists together with band II, the stretching vibration peak of C=O, and the stretching vibration peak of C-O described below), 1140–1178 cm^−1^ for the C-O stretching vibration peak; 966 cm^−1^ for (transform 1,4) -CH=CH- on the C-H out-of-plane bending vibration peak; and 910 cm^−1^ for (1,2-)—CH=CH_2_ on the C-H out-of-plane bending vibration peak. Among them, the absorption peak at 1738 cm^−1^ was formed by the superposition of C=O absorption in polyurethane and DOA. The absorption peaks at 1178 and 1140 cm^−1^ were formed by the superposition of C-O absorption peaks in polyurethane and DOA. The absorption peaks at 1535 and 1231 cm^−1^ represented the characteristic peaks of polyurethane hard segment urethane bonds, and the absorption peaks at 966 and 910 cm^−1^ represented the characteristic peaks of HTPB polymer. Using mass spectrometry, the gaseous products in the first weight loss stage of the HTPB films were determined to be CH_3_-containing gases, CO, CO_2_, N_2_O, and NO. During the second weight loss stage, the concentration of gas-containing CH_3_ increased significantly, whereas those of CO_2_ and CO decreased significantly.

Based on the decomposition peak temperatures of samples 6#, 7#, and 8# in the FTIR curves, the characteristic absorption peaks in the first stage were observed to have decreased; for example, the amide III band at 1231 cm^−1^ and the stretching vibration peak of C-O between 1140 and 1178 cm^−1^. As the sampling temperature increased, the gaseous products in the HTPB binder film contained CH_3_ gas, and the volatilisation of CO, CO_2_, N_2_O, and NO started. Furthermore, as the temperature increased, the DOA and TDI in the HTPB binder film were thermally decomposed and volatilised, leaving only those substances that were difficult to volatilise. When reheated, the substances that had not been completely volatilised in the first stage continued to volatilise; thus, the peak temperature in the first stage of weight loss decreased slightly. In contrast, reheating had almost no effect on the second weight loss stage.

**Figure 2:**
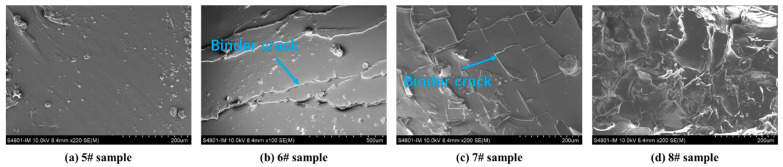
SEM morphology of the HTPB binder films heated to various temperatures.

SEM was used to analyse the apparent morphology of HTPB binder films at different sampling temperatures, as shown in Figure 2. It can be seen that the morphology of sample 5# is smooth and rich in viscoelasticity. Sample 6# begins to undergo changes, and as the components in the binder undergo thermal decomposition and volatilisation, the binder becomes brittle and cracks on the surface. As the sampling temperature reaches 180 °C, there are more cracks on the surface of sample 7#. When the sampling temperature is 220 °C, the 8# sample becomes more brittle, forming a bumpy surface.

#### 3.1.2. Thermal Decomposition of the HTPE Binder at Various Sampling Temperatures

**Figure 3:**
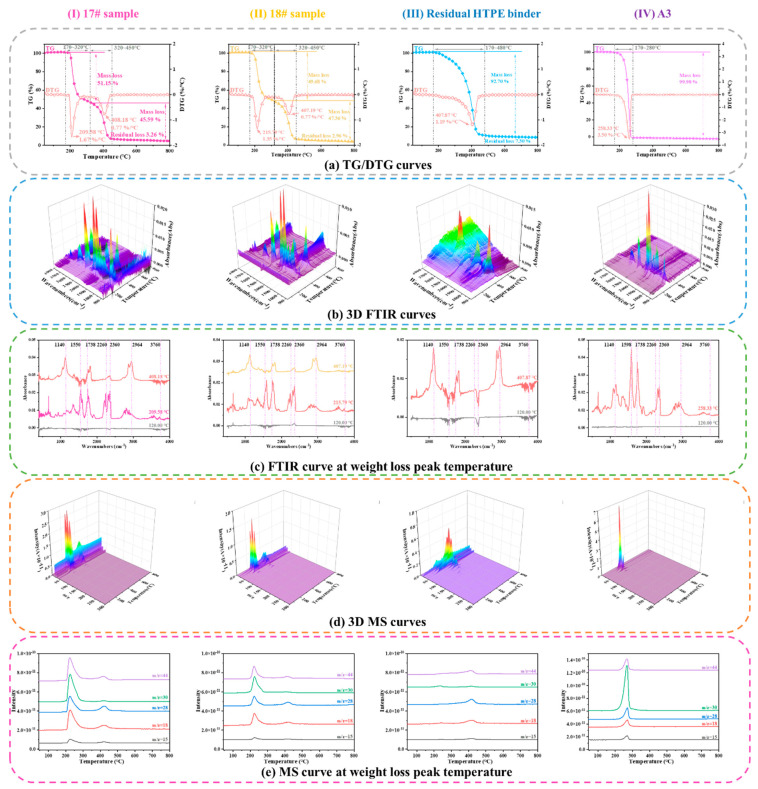
TG−FTIR−MS curves of the HTPE binder films heated to various temperatures.

Figure 3 shows the TG–FTIR–MS curves of the HTPE binder films heated to various temperatures. In Figure 3a, (I) and (II) show the TG/DTG curves of samples 17# and 18#, respectively. In Figure 3a, (III) shows the residual HTPE binder film samples after combustion, and (IV) shows the TG/DTG curves of component A3. The thermal weight loss process of the HTPE binder film can be observed to be completed in two stages. In the first stage, when the sampling temperature increases, the peak temperature of the weight loss of the HTPE binder film increases slightly, and the weight loss rate decreases slightly. In contrast, little effect is observed in the second weight loss stage. As the HTPE binder ignited before the temperature reached 180 °C, HTPE samples were not collected at temperatures exceeding this. Furthermore, the energetic plasticiser A3 was added to the HTPE binder film, which lowered the reaction temperature of the HTPE binder owing to the volatilisation and decomposition heat release of A3; A3 is observed to be completely volatilised between 150 °C and 269 °C.

Based on the positions of the main absorption peaks, their corresponding groups in the FTIR spectrum can be determined to be as follows: 2874–2964 cm^−1^ for C-H (where the peaks at 2964 and 2874 cm^−1^ correspond to the asymmetric and symmetric stretching vibration peaks, respectively, of C-H in CH_3_); 2260 cm^−1^ corresponding to N_2_O; 1738 cm^−1^ to the stretching vibration peak of C=O; and 1140–1178 cm^−1^ to the stretching vibration peak of C-O. Based on the intensity of the infrared absorption peak of the decomposition product, the decomposition product can be determined to be mainly composed of small molecular ethers, alkanes, and a small amount of aldehydes. Based on MS, the first stage of weight loss of HTPE can be determined to comprise mainly the pyrolysis of the A3 plasticiser, and the second stage of weight loss can be determined to comprise the pyrolysis of the HTPE polymer colloid.

Figure 1 and Figure 3 indicate that although the thermal decomposition process of both binder films is completed in two stages, the first weight loss decomposition peak temperature of the HTPE binder is 209.58 °C, whereas that of the HTPB binder is 294.92 °C, which is 85.34 °C higher than that of the HTPE binder. The second weight loss decomposition peak temperature of the HTPE binder is 408.18 °C, whereas that of the HTPB binder is 463.92 °C, 55.74 °C higher than that of the HTPE binder. Therefore, compared to HTPB binders, HTPE binders decompose more easily. Such different decomposition peak temperatures are bound to impact the thermal decomposition and cocombustion interactions of propellants.

**Figure 4:**
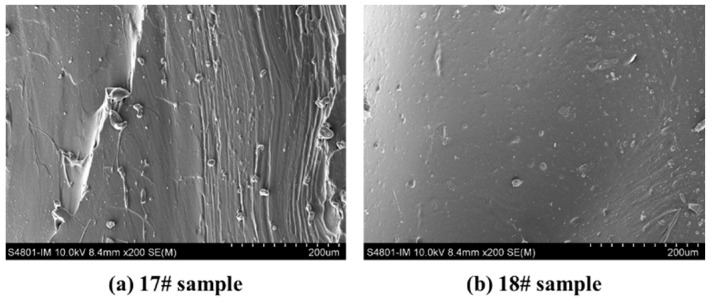
SEM morphology of the HTPE binder films heated to various temperatures.

SEM was used to analyse the apparent morphology of HTPE binder films at different sampling temperatures, as shown in Figure 4. It can be seen that the morphology of sample 17# is wrinkled and elastic. As the components in the binder undergo thermal decomposition and volatilisation, sample 18# begins to liquefy, making the binder more viscous and smoothing the surface wrinkles. It can be seen that as the sampling temperature increases, the HTPB binder begins to become brittle, while the HTPE binder becomes sticky, which may cause the HTPE binder to adhere to the surface of AP particles and affect the interaction between the two components.

#### 3.1.3. Thermal Decomposition of AP Particles at Various Sampling Temperatures

**Figure 5:**
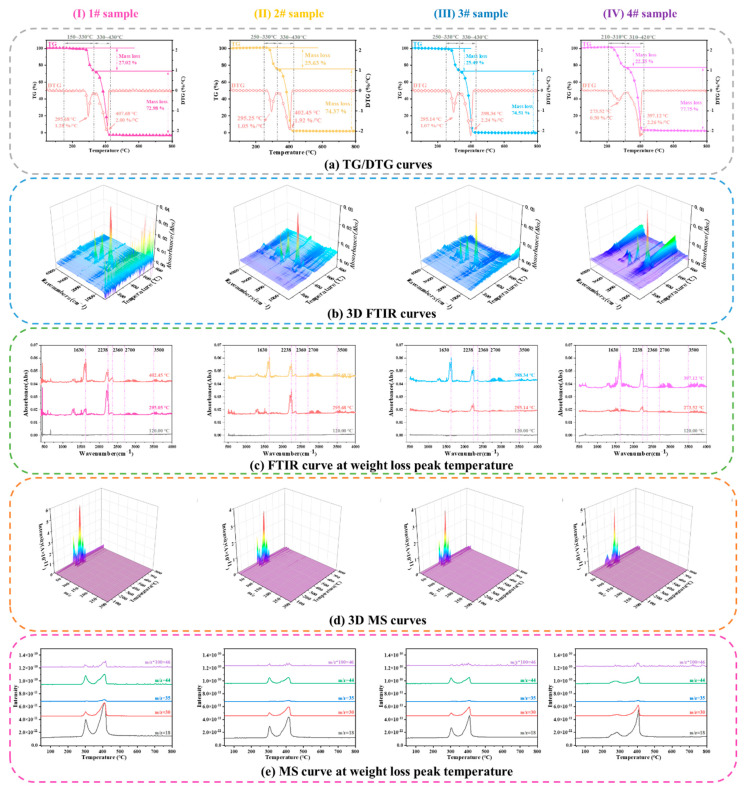
TG−FTIR−MS curves of the AP particles heated to various temperatures.

Figure 5 shows the TG–FTIR-MS curves of the AP particles heated to various temperatures. Figure 5a shows that two stages exist in the thermal weight loss process of AP particles: the low- and high-temperature weight loss stages. By increasing sampling temperature, the DTG low- and high-temperature decomposition peaks of AP advance slightly. Compared to sample 1#, the starts of the low-temperature decomposition of samples 2# and 3# are delayed because the sampling temperature consumes a portion of the defective AP nuclei during the period when the temperature is between 160 and 180 °C; therefore, the start of low-temperature decomposition is delayed during reheating. However, the low-temperature decomposition peak of sample 4# is observed 20 °C earlier than those of samples 2# and 3# because the AP particles generate pores, and the specific surface area increases when the sampling temperature is 220 °C, resulting in AP dissociation.

Based on the FTIR curves of the gaseous products decomposed from AP at the weight loss peak temperatures in Figure 5b,c, the wave numbers of N_2_O (2238 and 2201 cm^−1^), NO_2_ (1630 and 1598 cm^−1^), H_2_O (3500–4000 cm^−1^), and HCl (2700–3012 cm^−1^) can be determined by combining the data from (d) and (e). This analysis reveals that the main gaseous products of AP thermal decomposition are N_2_O and NO_2_.

It can be seen from Figure 5c that during the low-temperature weight loss stage, the NO_2_ absorption intensity of sample 1# is 0.0215, and the N_2_O absorption intensity is 0.0405. The NO_2_ absorption intensity of sample 2# is 0.0215, and the N_2_O absorption intensity is 0.0359. The NO_2_ absorption intensity of sample 3# is 0.0201, and the N_2_O absorption intensity is 0.0246. The NO_2_ absorption intensity of sample 4# is 0.0193, and the N_2_O absorption intensity is 0.0226. Their ratios are 1.88, 1.67, 1.22, and 1.17, respectively. As the sampling temperature increases, the absorption intensity ratio of NO_2_ and N_2_O gradually decreases during the low-temperature weight loss stage. During the high-temperature weight loss stage, the NO_2_ absorption intensity of sample 1# is 0.0594, and the N_2_O absorption intensity is 0.0514. The NO_2_ absorption intensity of sample 2# is 0.0608, and the N_2_O absorption intensity is 0.0560. The NO_2_ absorption intensity of sample 3# is 0.0604, and the N_2_O absorption intensity is 0.0530. The NO_2_ absorption intensity of sample 4# is 0.0640, and the N_2_O absorption intensity is 0.0511. Their ratios are thus 0.87, 0.92, 0.87, and 0.80, respectively. As the sampling temperature increases, the absorption intensity ratio of NO_2_ and N_2_O does not change significantly during the high-temperature weight loss stage.

**Figure 6:**
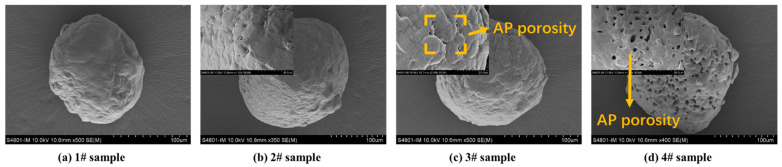
SEM morphology of the AP particles films heated to various temperatures.

SEM was used to analyse the apparent morphology of AP particles at different sampling temperatures, as shown in Figure 6. It can be seen that the surface of sample 1# is smooth and free from pores. Sample 2# began to undergo changes and the surface became uneven, but there were no pores. As the sampling temperature reaches 180 °C, there is a trend of increasing pores in sample 3#. When the sampling temperature is 220 °C, as the degree of decomposition deepens, sample 4# forms a porous AP structure.

Moreover, the changes in the intensity ratio of N_2_O to NO_2_ during the two weight loss stages indicate that a competitive relationship exists between the formation reaction of N_2_O and NO_2_ during the thermal decomposition of AP. This is consistent with the observations in reference [19]. The absorbance of N_2_O and NO_2_ during the low-temperature weight loss stage is greater than that of NO_2_, indicating that the products of N_2_O play a dominant role in the low-temperature weight loss process. However, during the high-temperature weight loss stage, the absorption intensity of NO_2_ is greater than that of N_2_O, indicating that the products of NO_2_ dominate high-temperature weight loss.

The first step of the thermal decomposition of AP is the dissociation of NH_4_ClO_4_ through proton transfer to form adsorbed NH_3_ and HClO_4_. Low-temperature thermal decomposition mainly occurs as a reaction between NH_3_ and HClO_4_ adsorbed onto the surface of the particles. At low temperatures, the decomposition products of HClO_4_ cannot oxidise NH_3_, and the remaining adsorbed NH_3_ covers the AP surface. When the particle surfaces are completely covered by NH_3_, low-temperature thermal decomposition causes a weight loss of approximately 30%.

The high-temperature thermal decomposition process of AP is mainly a gaseous-phase reaction: the adsorbed NH_3_ and HClO_4_ are pyrolysed and absorbed into the gaseous phase. In the gaseous phase, HClO_4_ further decomposes to generate oxidation products, whereas NH_3_ is oxidised by the oxidation products decomposed by HClO_4_ to generate the final products. As the sampling temperature increases, the severity of the partial decomposition of AP increases. When reheated, the specific surface area increases due to the presence of pores in the particles, and NH_4_ClO_4_ dissociation is more likely to occur, resulting in a decrease in the initial reaction temperature [31].

Although only 30% of AP decomposes at low temperatures, with the solid residue after decomposition is still AP, its physical properties change significantly and form a relatively stable porous material. However, when the temperature rises to 350–400 °C, AP undergoes high-temperature decomposition, releasing a large amount of energy. The thermal weight loss data of the HTPB and HTPE binders indicate that the first weight loss temperature of both HTPB and HTPE are low (less than 350 °C), whereas the second weight loss temperature exceeds 350 °C. Therefore, the two binders may interact at both the low- and high-temperature decomposition of AP.

### 3.2. Study on Cocombustion of Propellant Component

After analysing and understanding the thermal decomposition characteristics of individual components of propellant, the cocombustion and interaction are analysed and researched.

#### 3.2.1. Cocombustion of the HTPB Binder and AP Particles

**Figure 7:**
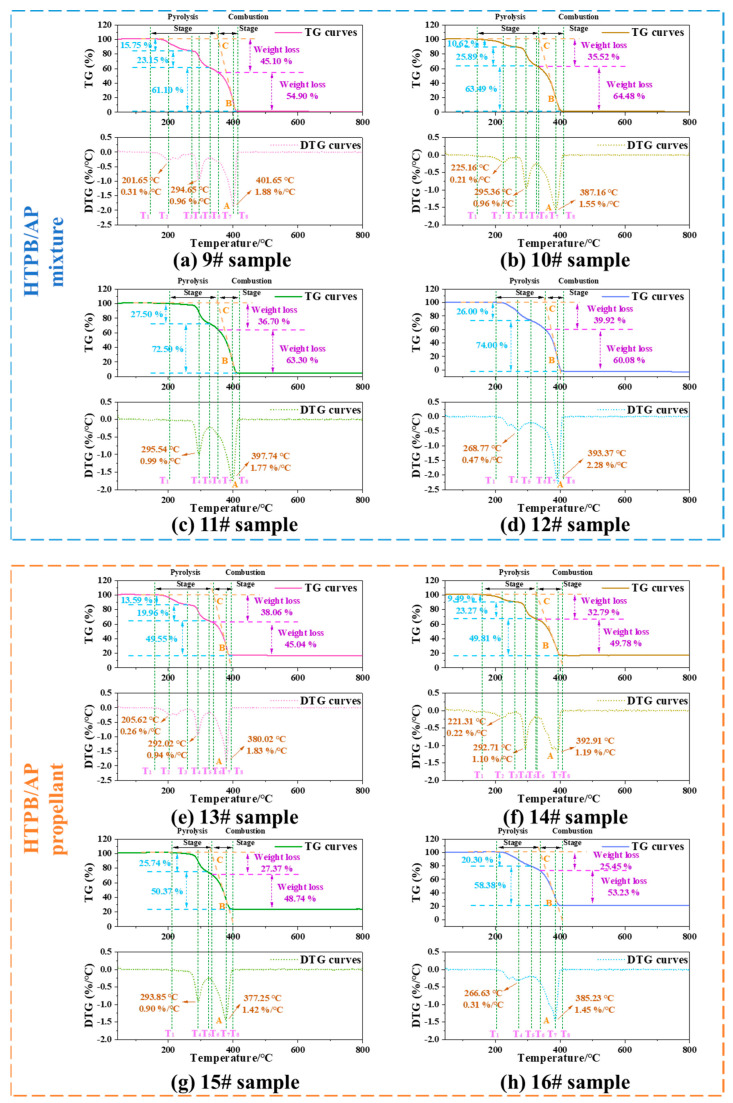
TG and DTG curves of the HTPB/AP mixtures and HTPB/AP/Al propellants at various sampling temperatures.

Figure 7 shows the TG and DTG curves of the HTPB/AP mixture and HTPB/AP/Al propellant, respectively. The thermal weight loss process of the original HTPB/AP mixture sample can be observed to be divided into three stages. The first stage is in the temperature range of 145–273 °C, with a maximum weight loss peak temperature at 201.65 °C and a gentle peak shape. The maximum weight loss rate is 0.31%/°C, and the weight loss is approximately 15.75%. This stage mainly results from the breakage and decomposition of the binder chain in HTPB. The second stage is in the temperature range of 273–330 °C, with a maximum peak temperature at 294.65 °C and a sharp peak shape. The weight loss is approximately 23.15%, and the maximum weight loss decomposition rate is 0.96%/°C. This stage consists mainly of the low-temperature decomposition of AP. The third stage is in the temperature range of 330–414 °C, with a peak temperature at 401.65 °C and a sharp peak shape. The maximum weight loss decomposition rate is 1.88%/°C, and the weight loss is approximately 61.10%. This stage consists mainly of the high-temperature decomposition of AP.

The thermal weight loss process of the HTPB/AP mixture samples heated to 160 °C can also be divided into three stages. The first stage is in the temperature range of 146–270 °C, with a maximum weight loss peak temperature at 225.16 °C and a gentle peak shape. The maximum weight loss rate is 0.21%/°C, and the weight loss is approximately 10.62%. This stage results mainly from the breaking and decomposition of the binder chain in HTPB. The second stage is in the temperature range of 270–328 °C, with a peak temperature at 295.36 °C and a sharp peak shape. The weight loss is approximately 25.89%, and the maximum weight loss decomposition rate is 0.96 /°C. This stage consists mainly of the low-temperature decomposition of AP. The third stage is in the temperature range of 328–408 °C, with a peak temperature at 387.16 °C and a sharp peak shape. The maximum weight loss decomposition rate is 1.55%/°C, and the weight loss is approximately 63.49%. This stage consists mainly of the high-temperature decomposition of AP.

The thermal weight loss process of the HTPB/AP mixture samples heated to 180 °C can be divided into two stages: the first stage is in the temperature range of 202–328 °C, with a peak temperature at 295.54 °C and a sharp peak shape. The maximum rate of weight loss is 0.99%/°C, and the weight loss is approximately 27.50%. This stage mainly results from the continued decomposition of the binder chain that is not completely decomposed in HTPB and the low-temperature decomposition of AP. The second stage is in the temperature range of 328–415 °C, with a peak temperature at 397.74 °C and a sharp peak shape. The maximum weight loss decomposition rate is 1.77%/°C, and the weight loss is approximately 72.50%. This stage consists mainly of the high-temperature decomposition of AP.

The thermal weight loss process of the HTPB/AP mixture sample heated to 220 °C can also be divided into two stages. The first stage is in the temperature range of 210–306 °C, with a maximum weight loss temperature at 268.77 °C and a gentle peak shape. The maximum weight loss rate is 0.47%/°C, and the weight loss is approximately 26.00%. This stage results mainly from the continued decomposition of the binder chain that is not fully decomposed in HTPB and the low-temperature decomposition of AP. The second stage is in the temperature range of 306–406 °C, with a peak temperature at 393.37 °C and a sharp peak shape. The maximum weight loss decomposition rate is 2.28%/°C, and the weight loss is approximately 74.00%. This stage consists mainly of the high-temperature decomposition of AP.

The thermal weight loss process of the original HTPB/AP/Al propellant sample can be divided into three stages. The first stage is in the temperature range of 149–265 °C, with a maximum weight loss peak temperature at 205.62 °C and a gentle peak shape. The maximum weight loss rate is 0.26%/°C, and the weight loss is approximately 13.59%. This stage results mainly from the breaking of the binder chain in HTPB. The second stage is in the temperature range of 265–326 °C, with a peak temperature at 292.02 °C and a sharp peak shape. The weight loss is approximately 19.96%, and the maximum weight loss decomposition rate is 0.94%/°C. This stage consists mainly of the low-temperature decomposition of AP. The third stage is in the temperature range of 326–391 °C, with a peak temperature at 380.02 °C and a sharp peak shape. The maximum weight loss decomposition rate is 1.83%/°C, and the weight loss is approximately 49.55%. This stage consists mainly of the high-temperature decomposition of AP.

The thermal weight loss process of the HTPB/AP/Al propellant samples heated to 160 °C can also be divided into three stages. The first stage is in the temperature range of 158–264 °C, with a maximum weight loss peak temperature at 221.31 °C and a gentle peak shape. The maximum weight loss rate is 0.22%/°C, and the weight loss is approximately 9.49%. This stage results mainly from the fracture and decomposition of the binder chain in HTPB. The second stage is in the temperature range of 264–325 °C, with a peak temperature at 292.71 °C and a sharp peak shape. The weight loss is approximately 23.27%, and the maximum weight loss decomposition rate is 1.10%/°C. This stage consists mainly of the low-temperature decomposition of AP. The third stage is in the temperature range of 325–401 °C, with a peak temperature at 392.91 °C and a sharp peak shape. The maximum weight loss decomposition rate is 1.19%/°C, and the weight loss is approximately 49.81%. This stage consists mainly of the high-temperature decomposition of AP.

The thermal weight loss process of the HTPB/AP/Al propellant samples heated to 180 °C can be divided into two stages. The first stage is in the temperature range of 197–324 °C, with a peak temperature at 293.85 °C and a sharp peak shape. The maximum rate of weight loss is 0.90%/°C, and the weight loss is approximately 25.74%. This stage results mainly from the continued decomposition of the binder chain that is not fully decomposed in HTPB and the low-temperature decomposition of AP. The second stage is in the temperature range of 324–399 °C, with a peak temperature at 377.25 °C and a sharp peak shape. The maximum weight loss decomposition rate is 1.42%/°C, and the weight loss is approximately 0.37%. This stage consists mainly of the high-temperature decomposition of AP.

The thermal weight loss process of the HTPB/AP/Al propellant samples heated to 220 °C can also be divided into two stages. The first stage is in the temperature range of 206–310 °C, with a maximum peak temperature at 266.63 °C and a gentle peak shape. The maximum rate of weight loss is 0.31%/°C, and the weight loss is approximately 20.30%. This stage results mainly from the continued decomposition of the binder chain that is not completely decomposed in HTPB and the low-temperature decomposition of AP. The second stage is in the temperature range of 310–400 °C, with a peak temperature at 385.23 °C and a sharp peak shape. The maximum weight loss decomposition rate is 1.45%/°C, and the weight loss is approximately 58.38%. This stage consists mainly of the high-temperature decomposition of AP.

When the sampling temperature is 180 °C and 220 °C, the thermal decomposition process can be divided into two stages. However, it can be divided into three stages when unheated and when the sampling temperature is 160 °C. This is because the binder in the mixture decomposes when the temperature exceeds 180 °C, meaning that a decomposition stage of the binder is lacking. This is because during the preparation of the sample, the first stage of binder decomposition has been completed. Therefore, it made the first-stage and second-stage original decomposition temperature higher.

**Figure 8:**
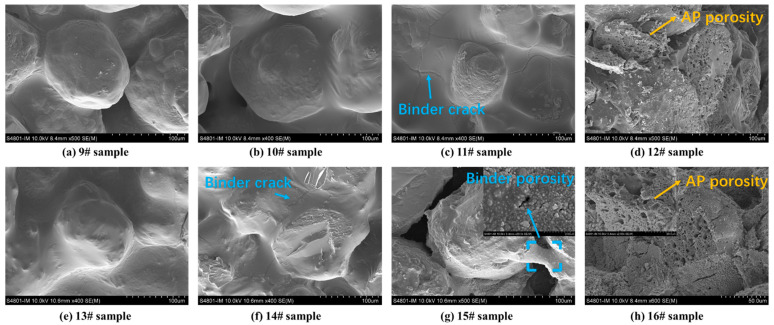
SEM morphology of the HTPB/AP mixtures and HTPB/AP/Al propellants heated to various temperatures.

SEM was used to analyse the apparent morphology of HTPB/AP mixtures and HTPB/AP/Al propellants at different sampling temperatures, as shown in Figure 8. It can be seen that as the sampling temperature increases, cracks begin to appear in the adhesive of sample 11# at 180 °C, while cracks have already appeared in sample 14# at 160 °C. It indicates that the adhesive has decomposed at this time, and in sample 15#, it can be seen that the adhesive is filled with pores while AP particles have no detailed changes. This also indicates that the adhesive decomposes first during the heating process. In samples 12# and 16#, pores are observed in AP particles. Both the decomposition of the binder and the pore structure of AP particles will have an impact on the combustion of the propellant.

The characteristic temperature is an important characteristic parameter in the heating process of propellants. As shown in Figure 7, T_1_ is the temperature at which the propellant begins to decompose, T_2_ is the temperature corresponding to the first peak of the weight loss rate, T_3_ is the end temperature of the first stage of weight loss, T_4_ is the temperature corresponding to the second peak of the weight loss rate, T_5_ is the end temperature of the second stage of weight loss, T_6_ is the temperature at which the propellant begins to burn (ignition temperature), T_7_ is the temperature corresponding to the third peak of the weight loss rate, and T_8_ is the temperature at which all combustible elements in the propellant are burned out. The ignition temperature is defined as the temperature corresponding to the intersection point C of the TG baseline and the tangent line of the TG descent point B corresponding to the peak point A on the DTG curve [32,33,34]. The heating process of the propellant can be mainly divided into two stages: the first stage is thermal decomposition and the second stage is combustion after ignition. Understanding the cocombustion behaviour of propellant components is important for investigating the interactions between the propellant components.

Figure 7 shows that the heating weight loss process of the propellant can be divided into two stages: the thermal decomposition before ignition of the propellant and the combustion stage after ignition. The first weight loss stage of the HTPB/AP mixture is characterised by a slow weight loss, which is 35% to 45% higher than the binder content in the propellant. This indicates that the first stage results not only from the thermal decomposition of the binder but also from the low-temperature decomposition of AP particles. The second stage is characterised by a rapid weight loss of 55% to 65%, mainly owing to the combustion of AP oxidants in the propellant. The weight loss of the HTPB/AP/Al propellant in the first stage is 32% to 40%, which is also higher than the binder content in the propellant. The second stage includes the thermal decomposition of the binder and the low-temperature decomposition of the AP particles. The weight loss in the second stage is 45% to 53%. The material remaining after the second stage consists of Al powder and a reaction residue.

To analyse the combustion characteristics of the propellants comprehensively, the flammability index, *S*, is defined as follows [35].

The combustion at lower heating rates can be determined by chemical reaction kinetics. According to Arrhenius’ law,

(1) $$\frac{dW}{dt}=Aexp\left(-\frac{E}{RT}\right),$$

where *dW/dt* is the combustion rate (%/°C), *A* is the pre-exponential factor (min^−1^), *E* is the activation energy (kJ/mol), and *T* is the temperature (K).

From Equation (1), the following derivation can be obtained:

(2) $$\frac{R}{E}\frac{d}{dT}\left(\frac{dW}{dt}\right)=\frac{dW}{dt}\frac{1}{T^{2}}.$$

At the ignition temperature, the aforementioned formula becomes

(3) $$\frac{R}{E}\frac{d}{dT}{\left(\frac{dW}{dt}\right)}_{T=T_{i}}={\left(\frac{dW}{dt}\right)}_{T=T_{i}}\frac{1}{T_{i}^{2}}.$$

Equation (3) can be converted as follows:

(4) $$\frac{R}{E}\frac{d}{dT}{\left(\frac{dW}{dt}\right)}_{T=T_{i}}\frac{{\left(\frac{dW}{dt}\right)}_{max}}{{\left(\frac{dW}{dt}\right)}_{T=T_{i}}}\frac{{\left(\frac{dW}{dt}\right)}_{mean}}{T_{h}}=\frac{{\left(\frac{dW}{dt}\right)}_{max}{\left(\frac{dW}{dt}\right)}_{mean}}{T_{i}^{2}T_{h}},$$

where (*dW/dt*)*_max_* is the maximum combustion rate (%/°C), (*dW/dt*)*_mean_* is the average combustion rate (%/°C), (*dW/dt*)*_T=Ti_* is the combustion rate at the ignition temperature (%/°C), T_i_ is the ignition temperature (°C), and *T_h_* is the burnout temperature (°C). *R/E* represents the reactivity of the propellant: the greater the value, the faster the reaction speed. At the ignition temperature, *d/dT* (*dW/dt*)*_T=Ti_* is the percentage of combustion rate conversion: the greater the value, the more rapid the ignition. Moreover, at the ignition temperature, (*dW/dt*)*_max_*/(*dW/dt*)*_T=Ti_* is the ratio of the maximum combustion rate to the combustion rate. Furthermore, (*dW/dt*)*_mean_*/*T_H_* represents the ratio of the average combustion rate to the burnout temperature: the greater the value, the faster the propellant burns. The product of the aforementioned terms reflects the combustion characteristics of the propellant, and its flammability index *S* is defined as

(5) $$S=\frac{{\left(\frac{dW}{dt}\right)}_{max}{\left(\frac{dW}{dt}\right)}_{mean}}{T_{i}^{2}T_{h}}.$$

Here, *T_i_* and *T_h_* are *T*_6_ and *T*_8_, respectively. The calculated flammability indices of the propellant samples are listed in Table 3.

Table 3 shows that the *S* index of sample 9# is 3.34 × 10^−8^, and *S* decreases to 2.77 × 10^−8^ as the sampling temperature increases to 160 °C. This occurs because the HTPB binder in the propellant is thermally decomposed, thus weakening the interaction between the binder and AP particles. As the sampling temperature continues to increase, the *S* index gradually increases to 4.24 × 10^−8^; although the HTPB binder has decomposed and the interaction is weak, pores are generated inside the sample at that time, which, in return, increases the specific surface area. To put it another way, even the interaction is weak, while the increase in the specific surface area could also enlarge the interaction effect. Therefore, the combustion characteristics are first mild and then become intense. The HTPB/AP/Al propellants exhibit the same trend as the mixtures, indicating that the addition of the Al powder has no significant effect on the interaction between the binder and AP other than performing a catalytic role.

#### 3.2.2. Cocombustion of the HTPE Binder and AP Particles

**Figure 9:**
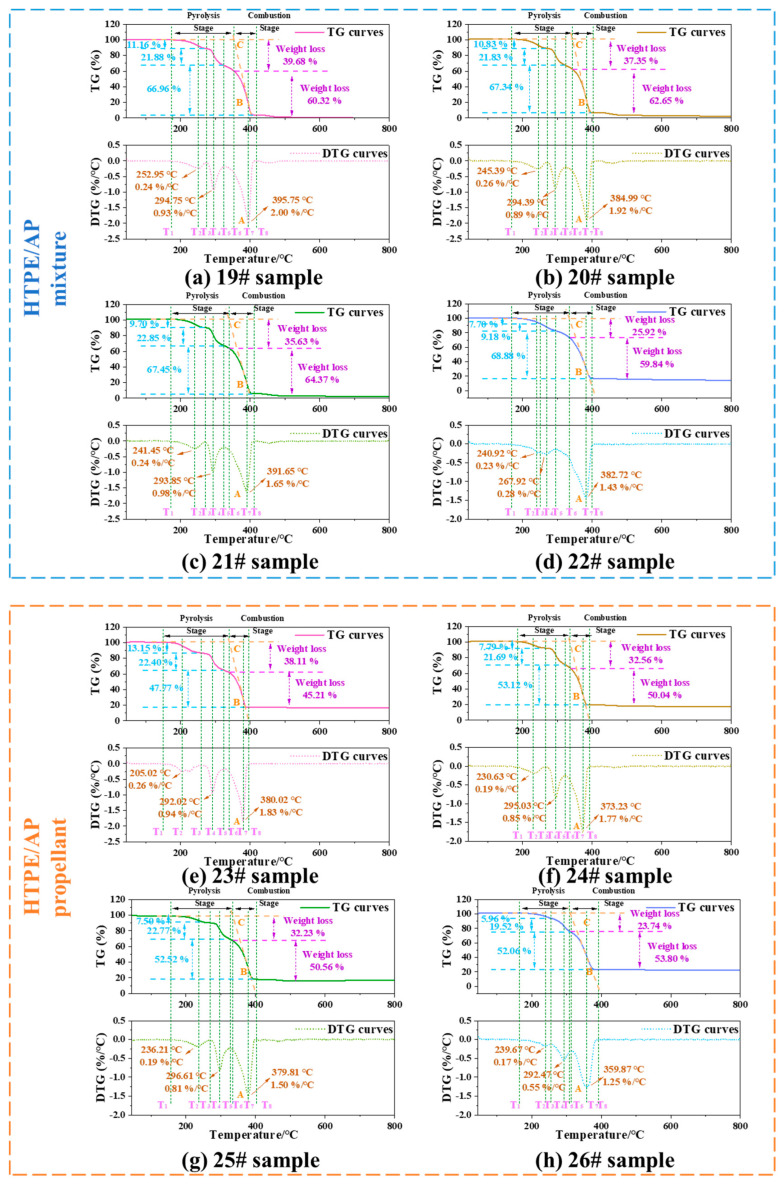
TG and DTG curves of the HTPE/AP mixtures and HTPE/AP/Al propellants at various sampling temperatures.

Figure 9 shows the TG and DTG curves of the HTPE/AP mixture and HTPE/AP/Al propellant, respectively. The thermal weight loss process of the original HTPE/AP mixture sample and samples 20#, 21#, and 22# can all be observed to be divided into three stages.

For the original HTPE/AP mixture sample, the first stage is in the temperature range of 174–274 °C, with a maximum weight loss peak temperature at 252.95 °C and a gentle peak shape. The maximum weight loss rate is 0.24%/°C, and the weight loss is approximately 11.16%. This stage results mainly from the breaking and decomposition of the binder chain in HTPE. The second stage is in the temperature range of 274–327 °C, with a maximum peak temperature at 294.75 °C and a sharp peak shape. The weight loss is approximately 21.88%, and the maximum weight loss decomposition rate is 0.93%/°C. This stage consists mainly of the low-temperature decomposition of AP. The third stage is in the temperature range of 327–411 °C, with a peak temperature at 395.75 °C and a sharp peak shape. The maximum weight loss decomposition rate is 2.00%/°C, and the weight loss is approximately 66.96%. This stage consists mainly of the high-temperature decomposition of AP.

For sample 20#, the first stage is in the temperature range of 173–270 °C, with a maximum weight loss peak temperature at 245.39 °C and a gentle peak shape. The maximum weight loss rate is 0.26%/°C, and the weight loss is approximately 10.83%. The second stage is in the temperature range of 270–324 °C, with a maximum peak temperature at 294.39 °C and a sharp peak shape. The weight loss is approximately 21.83%, and the maximum weight loss decomposition rate is 0.89%/°C. Finally, the third stage is in the temperature range of 324–399 °C, with a peak temperature at 384.99 °C and a sharp peak shape. The maximum weight loss decomposition rate is 1.92%/°C, and the weight loss is approximately 67.34%

For sample 21#, the first stage is in the temperature range of 173–270 °C, with a maximum weight loss peak temperature at 241.45 °C and a gentle peak shape. The maximum weight loss rate is 0.24%/°C, and the weight loss is approximately 9.70%. The second stage is in the temperature range of 270–324 °C, with a peak temperature at 293.85 °C and a sharp peak shape. The weight loss is approximately 22.85%, and the maximum weight loss decomposition rate is 0.98%/°C. The third stage is in the temperature range of 324–408 °C, with a peak temperature at 391.65 °C and a sharp peak shape. The maximum weight loss decomposition rate is 1.65%/°C, and the weight loss is approximately 67.45%.

For sample 22#, the first stage is in the temperature range of 171–251 °C, with a maximum weight loss peak temperature at 240.92 °C, maximum weight loss rate of 0.23%/°C, and weight loss of approximately 7.70%. The second stage is connected to the first stage, with a weight loss of approximately 9.18% in the temperature range of 251–293 °C. The third stage is in the temperature range of 293–405 °C, with a peak temperature at 382.72 °C and a sharp peak shape. The maximum weight loss decomposition rate is 1.43%/°C, and the weight loss is approximately 68.88%.

On account of the defects on the AP crystal surface, a small number of AP molecules at the defect sites readily dissociate into NH_3_ and HClO_4_ via proton transfer at lower temperatures. Furthermore, as a strong acid, HClO_4_ readily reacts with the oxygen atoms of the ether bond in the HTPE molecular chain to form a salt, which makes the thermal stability of the ether bond decrease. Therefore, the earlier initial decomposition temperature of the first decomposition stage of the HTPE/AP mixture may have been caused by the small number of AP decomposition products promoting the decomposition of the HTPE binder.

The thermal weight loss process of the original HTPE/AP/Al propellant sample and samples 24#, 25#, and 26# can all be divided into three stages. For the original HTPE/AP/Al propellant sample, the first stage is in the temperature range of 136–259 °C, with a maximum weight loss peak temperature at 205.02 °C and a gentle peak shape. The maximum weight loss rate is 0.26%/°C, and the weight loss is approximately 13.15%. This stage results mainly from the breaking of the binder chains in HTPE. The second stage is in the temperature range of 259–326 °C, with a peak temperature at 292.02 °C and a sharp peak shape. The weight loss is approximately 22.40%, and the maximum weight loss decomposition rate is 0.94%/°C. This stage consists mainly of the low-temperature decomposition of AP. The third stage is in the temperature range of 326–392 °C, with a peak temperature at 380.02 °C and a sharp peak shape. The maximum weight loss decomposition rate is 1.83%/°C, and the weight loss is approximately 47.77%. This stage consists mainly of the high-temperature decomposition of AP.

For sample 24#, the first stage is in the temperature range of 170–270 °C, with a maximum weight loss peak temperature at 230.03 °C and a gentle peak shape. The maximum weight loss rate is 0.19%/°C, and the weight loss is approximately 7.79%. The second stage is in the temperature range of 270–324 °C, with a peak temperature at 295.03 °C and a sharp peak shape. The weight loss is approximately 21.69%, and the maximum weight loss decomposition rate is 0.85%/°C. The third stage is in the temperature range of 324–391 °C, with a peak temperature at 373.23 °C and a sharp peak shape. The maximum weight loss decomposition rate is 1.77%/°C, and the weight loss is approximately 53.12%. This stage consists mainly of the high-temperature decomposition of AP.

For sample 25#, the first stage is in the temperature range of 167–270 °C, with a maximum weight loss peak temperature at 236.21 °C and a gentle peak shape. The maximum weight loss rate is 0.19%/°C, and the weight loss is approximately 7.50%. The second stage is in the temperature range of 270–325 °C, with a maximum peak temperature at 296.61 °C and a sharp peak shape. The weight loss is approximately 22.77%, and the maximum weight loss decomposition rate is 0.81%/°C. The third stage is in the temperature range of 325–400 °C, with a peak temperature at 379.81 °C and a sharp peak shape. The maximum weight loss decomposition rate is 1.50%/°C, and the weight loss is approximately 52.52%.

For sample 26#, the first stage is in the temperature range of 164–255 °C, with a maximum weight loss peak temperature at 239.67 °C, a maximum weight loss rate of 0.17%/°C, and a weight loss of approximately 5.96%. The second stage is in the temperature range of 255–313 °C, with a peak temperature at 292.47 °C and a sharp peak shape. The weight loss is approximately 19.52%, and the maximum weight loss decomposition rate is 0.55%/°C. The third stage is in the temperature range of 313–390 °C, with a peak temperature at 359.87 °C and a sharp peak shape. The maximum weight loss decomposition rate is 1.25%/°C, and the weight loss is approximately 52.06%.

In contrast with the HTPB/AP mixture, the thermal decomposition processes of all the aforementioned samples can be divided into three stages. The addition of Al power will not affect its decomposition process.

**Figure 10:**
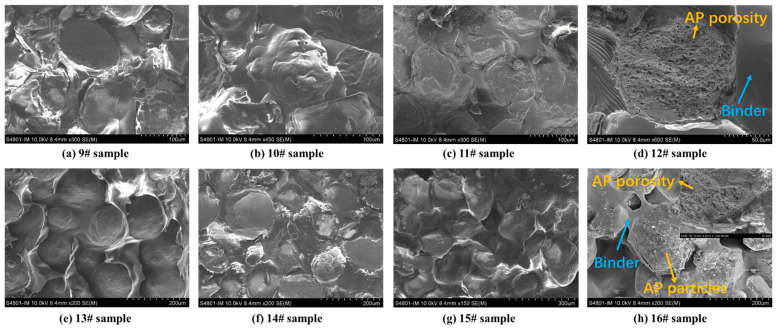
SEM morphology of the HTPE/AP mixtures and HTPE/AP/Al propellants heated to various temperatures.

SEM was used to analyse the apparent morphology of HTPE/AP mixtures and HTPE/AP/Al propellants at different sampling temperatures, as shown in Figure 10. It can be seen that in the HTPE/AP mixture and HTPE/AP/Al propellant, as the sampling temperature increases, the HTPE binder gradually liquefies and coats the surface of AP particles. From samples 12# and 16#, it can be seen that the HTPE binder at this time is still in a viscoelastic state, but the AP particles have already decomposed.

It can be seen that the interactions between the components of the two binder systems are different. This is because the HTPB binder becomes harder and more brittle as the sample temperature increases, causing it to debond from the AP particles, which in turn weakens the interactions between them. The HTPE binder becomes softer and reaches a certain degree of liquefaction, while continuing to adhere to the AP particles and interact with them.

Figure 9 shows the TG/DTG curves and characteristic temperatures of the HTPE/AP mixtures and HTPE/AP/Al propellants at various temperatures. The heating weight loss processes of the propellants can be divided into two stages. The first stage of the HTPE/AP mixture exhibits a slow weight loss, with a total weight loss of 25–40%, which is higher than the binder content in the propellant, indicating that this stage involves not only the thermal decomposition of the binder but also the low-temperature decomposition of the AP particles. The rapid weight loss in the second stage results mainly from the combustion of the AP oxidant in the propellant, and the weight loss in this stage is approximately 60%. The weight loss of the HTPE/AP/HTPB propellant in the first stage is between 23% and 38%, which is also higher than the binder content in the propellant. This stage includes the thermal decomposition of the binder and the low-temperature decomposition of the AP particles. The weight loss in the second stage is between 45% and 53%. The material that remains after the second stage consists of Al powder and a reaction residue.

Table 4 shows that the *S* index of sample 19# is 3.78 × 10^−8^, and *S* increases to 4.05 × 10^−8^ as the sampling temperature increases to 160 °C. This occurs because as the sampling temperature increases, the HTPE binder liquefies under heat and adheres more tightly to the AP particles to coat their surfaces, resulting in stronger interactions. However, as the sampling temperature increases, *S* gradually decreases to 2.78 × 10^−8^ owing to the decomposition of the energetic plasticiser A3 in the binder, which cannot provide the heat released by its decomposition to accelerate the low-temperature decomposition of AP. Therefore, the combustion characteristics are initially violent and then slow. The HTPE/AP/Al propellant exhibits the same trend as the mixture, indicating that the addition of Al powder has no significant effect on the interaction between the binder and AP other than performing a catalytic role.

### 3.3. Study of the Interaction of Propellant Component

#### 3.3.1. Interaction between the HTPB Binder and AP Particles

To investigate whether there is an interaction between the binder and AP particles, the theoretical TG/DTG curve of the blend was calculated from the average weight of the individual as follows:

(6) $$W=\alpha W_{binder}+\beta W_{AP}$$

where *W_binder_* and *W_AP_* are the weight loss rates of the binder and AP particles, respectively, and *α* and *β* are their respective proportions in the propellant.

**Figure 11:**
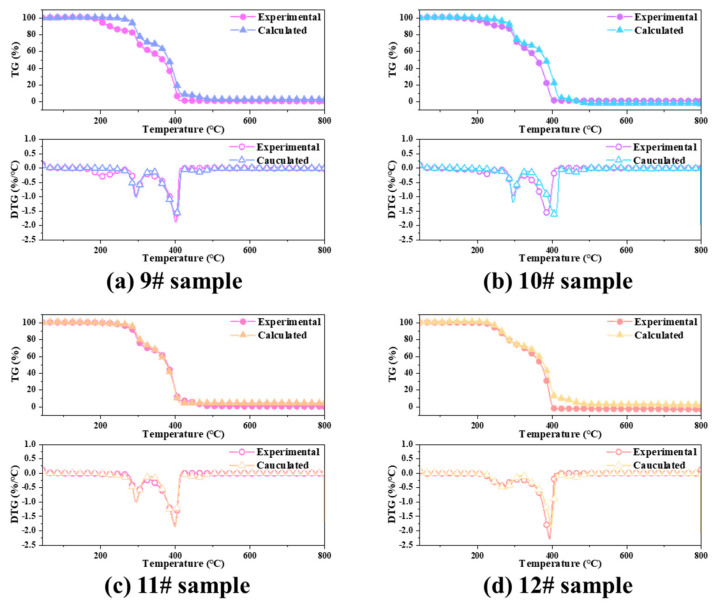
Comparison between the experimental and calculated for HTPB/AP mixture TG and DTG curves.

**Figure 12:**
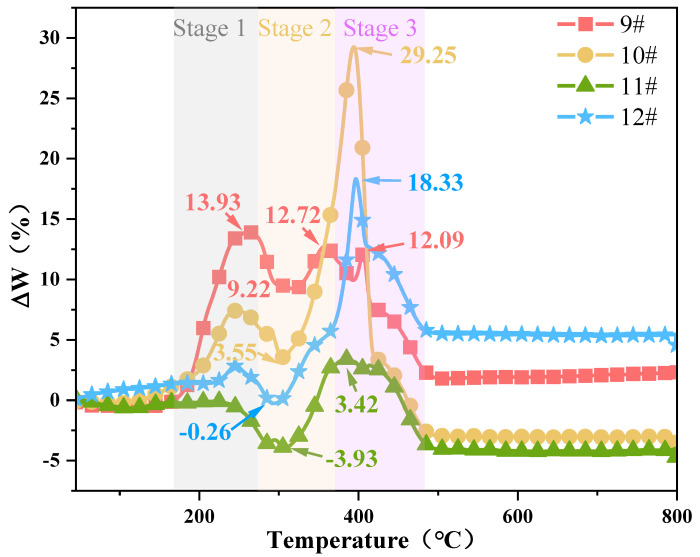
Difference between the HTPB/AP mixture experimental and theoretical weight loss.

The theoretical thermogravimetric curve for the binder mass ratio to AP particles at 18:82 was calculated. The experimental and calculated TG curves are shown in Figure 11. To further clarify the interaction between the HTPB binder and AP particles, ΔW (ΔW = TG_calculated_ − TG_empirical_) is defined as the difference in weight loss. Figure 12 shows the composition of the HTPB/AP mixture as ΔW changes with temperature.

The calculated DTG curve of the HTPB/AP mixture almost coincides with the experimental DTG curve within the temperature range below 150 °C. When the temperature of all samples exceeds 150 °C, the calculated TG curve lags behind the experimental TG curve. All the interactions between HTPB and the AP particles are positive and occur at all stages. At 200 °C, the calculated maximum weight loss is 0.30%/°C higher than the experimental value, indicating that the HTPB binder and AP particles interact at low temperatures. Compared with the calculated DTG curve, the experimental DTG curve shifts in the 260–420 °C region. This further confirms the significant interaction between the HTPB binder and AP particles.

Three maximum peaks exist in sample 9#, notably at 262 °C, with a deviation value as high as 13.93. The deviations are 12.72 and 12.09 at 357 and 405 °C, respectively. The maximum deviation for samples 10#, 11#, and 12# are 29.25, 3.42, and 18.33, respectively. These deviations indicate that a promoted interaction occurs between the HTPB binder and AP particles during both the thermal decomposition and combustion stages. This can be attributed to the exothermic heating effect of the HTPB decomposition process, which causes the AP to dissociate at low temperatures and release highly oxidising products in advance. The effect is more significant during the thermal decomposition stage of the unheated HTPB/AP mixture. Above 500 °C, ΔW is stable due to the combustion process of the blend being almost complete.

**Figure 13:**
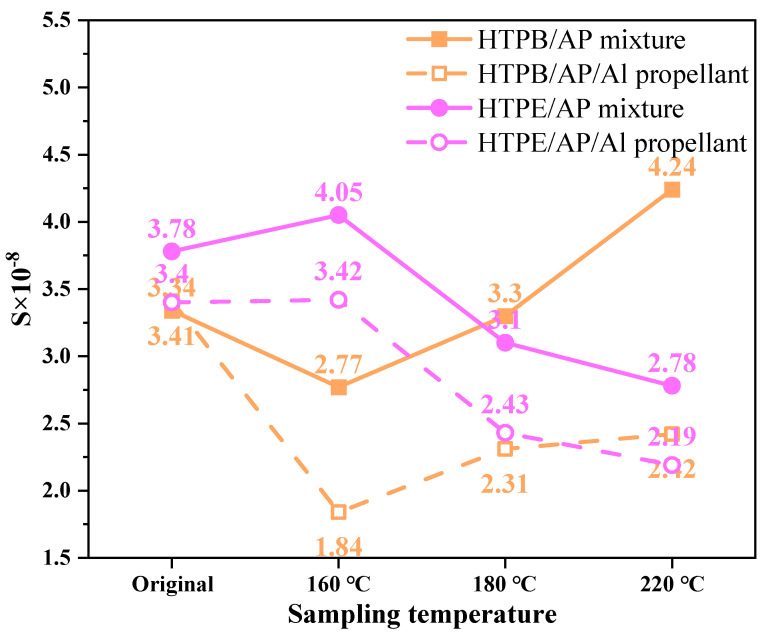
Combustion characteristic index *S* of HTPB and HTPE binder system.

The interaction between the HTPB binder and AP differs from the mixed HTPB/AP system. Figure 13 shows the combustion characteristic index *S* of the HTPB and HTPE binder systems. As can be seen from Figure 12 and Figure 13, the impact of ΔW is divided into three stages, which have varying degrees of impact. The first weight loss stages of samples 9#, 10#, and 11# show a gradually decreasing ΔW, and as the sampling temperature increases, the HTPB binder gradually decomposes. Thus, the AP particles promote the decomposition of the HTPB binder at this stage. The second weight loss stage also shows a gradually decreasing ΔW, but the interaction is weaker than that in the first weight loss stage, and ΔW has a negative promoting effect. In the third weight loss stage, ΔW first increases and then decreases as the sampling temperature increases. This is due to the HTPB binder gradually decomposing while weight loss continues, leaving behind substances that are difficult to decompose, thus gradually weakening the interaction. However, the combustion characteristic index, *S*, of sample 12# is the largest, and the interaction between the HTPB binder and AP particles is the largest in the third stage. The main interaction between the HTPB binder and AP particles occurs in the combustion stage. The interaction of various components in the mixed HTPB/AP system is not only related to its thermal decomposition stage but also affected by its decomposition products and many other factors, such as pore structure.

#### 3.3.2. Interaction between the HTPE Binder and AP Particles

**Figure 14:**
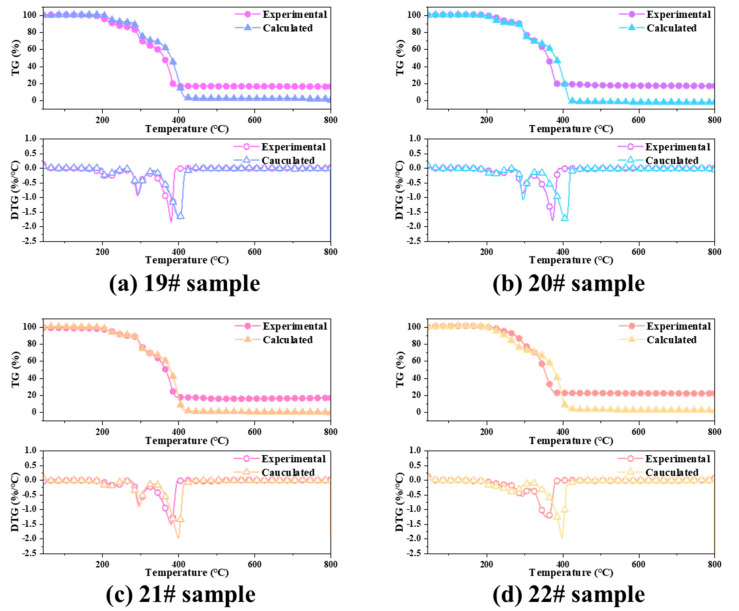
Comparison between the experimental and calculated for HTPE/AP mixture TG and DTG curves.

**Figure 15:**
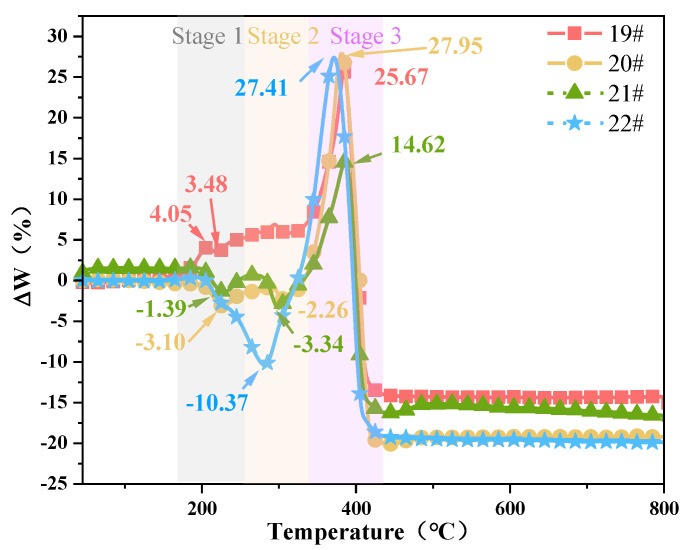
Difference between the HTPE/AP mixture experimental and theoretical weight loss.

The experimental and calculated TG curves of the HTPE/AP mixture at various sampling temperatures are shown in Figure 14 (the theoretical calculated values in samples 21# and 22# were calculated by using sample 18#). Figure 15 shows the composition of the HTPE/AP mixture at various sampling temperatures, and ΔW changes along with temperature. It can be seen that when the temperature is below 160 °C, the calculated DTG curve of the HTPE/AP mixture is almost consistent with the experimental DTG curve. When the temperature exceeds 160 °C, the calculated TG curve of sample 19# lags behind the experimental TG curve. When the temperature is between 150 and 320 °C, the experimental TG curves of samples 20#, 21#, and 22# lag behind the calculated TG curve. When the temperature exceeds 320 °C, the calculated TG curves of 20#, 21#, and 22# lag behind the experimental TG curve. There are three peaks in samples 19#, 20#, and 21#, of which sample 19# has a value of 4.05 at 206 °C and 3.48 at 220 °C, and a deviation value of up to 25.67 at 385 °C. Sample 20# is −3.10 at 226 °C, −2.26 at 304 °C, and 27.95 at 381 °C. Sample 21# is −1.39 at 229 °C, −3.34 at 299 °C, and 14.62 at 386 °C. Sample 22# has two peaks, ranging from −10.37 at 281 °C to 27.41 at 372 °C. These deviations indicate that when unheated, a positive promoting effect exists between HTPE and the AP particles, whereas when the sampling temperature exceeds 160 °C, a blocking effect exists between HTPE and the AP particles in the first and second weight loss stages of the HTPE/AP mixture and a positive promoting effect in the third weight loss stage. The main interaction between the HTPE binder and AP particles is in the thermal decomposition stage.

As can be seen from Figure 13 and Figure 15, the impact of ΔW is divided into three stages; yet, these three stages have varying degrees of impact, while the third stage has the least. The interaction between the binder and AP is different in the mixed system.

The unheated HTPE binder was beneficial to the low- and high-temperature decompositions of AP. This is related to the structural and thermal decomposition characteristics of HTPE. When AP is promoted, the HTPE binder can decompose in advance to produce short-chain polyethers. Moreover, as the temperature increases, the HClO_4_ produced by decomposition consumes a large amount of the HTPE binder owing to its decomposition and participation in oxidation reactions. However, when the HTPE propellant is heated, the short-chain polyether produced by the partial decomposition of the binder in it will fill the holes formed by the decomposition of the AP surface, thus acting as a coating and insulation, slowing down further decomposition of AP and inhibiting the concentrated and rapid release of decomposition heat. Therefore, the heated HTPE/AP mixture has a blocking effect, which can be achieved from the first stage when a decrease in ΔW is observed. It can be seen that the interaction of various elements in the mixed HTPE/AP system is not only related to its thermal decomposition stage but also affected by the binder decomposition products and many other factors, such as pore structure. Furthermore, it is also very beneficial for solid propellants to slow down the reaction of the AP oxidation products with Al powder under heating conditions, thereby reducing the responsiveness of solid propellants.

## 4. Conclusions

By studying the interactions between the components of two binder systems at various temperatures, the conclusions are as follows:

(1) The first and second weight loss decomposition peak temperatures of the HTPB binder are 85.34 and 55.74 °C higher, respectively, than those of the HTPE binder. Therefore, compared to the HTPB binders, the HTPE binders are more easily decomposed.
(2) As the sampling temperature increases, the S index of the HTPB/AP mixture initially decreases from 3.34 × 10^−8^ to 2.77 × 10^−8^, then increases to 4.24 × 10^−8^, indicating that its combustion characteristics are initially mild and then intensify. In contrast, the S index of the HTPE/AP mixture from 3.78 × 10^−8^ first increases to 4.05 × 10^−8^, then decreases to 2.78 × 10^−8^, indicating that its combustion characteristics are initially rapid and then slow down.
(3) The ΔW deviation between the heated HTPB binder and AP particles is positive, and the maximum deviations are 13.93, 29.25, 3.42, and 18.33, respectively. This indicates a promoting interaction between the HTPB binder and AP particles during the thermal decomposition and combustion stages. The ΔW deviation between the heated HTPE binder and AP particles is negative in the first and second weight loss stages, but positive in the third weight loss stage, with maximum deviations of 25.67, 27.95, 14.62, and 27.41, respectively. During the first and second weight loss stages of the HTPE/AP mixture, there is a blocking effect between the HTPE and AP particles on the surface, and a positive promoting effect appears in the third weight loss stage. The main interaction between the HTPE binder and AP particles occurs in the thermal decomposition stage.

The study of the interaction of the propellant component after heating is an important influencing factor for mastering and understanding the slow burning mechanism and response severity of propellants. In addition, the influence of the microstructure of propellants after heating cannot be ignored, and it is significant for comprehensively understanding the thermal safety of propellants.

## Figures and Tables

**Table 1:** Composition of the sample formula (wt.%).

System	Sample	HTPB	DOA ^a^	TDI ^b^	AP	Al	
HTPB system	HTPB binder	42.00	54.67	3.33			
	AP				100.00		
	HTPB/AP mixture	7.56	9.84	0.60	82.00		
	HTPB/AP/Al propellant	HTPB 6.30	DOA 8.20	TDI 0.50	AP 68.00	Al 17.00	
		HTPE	A3 ^c^	IPDI ^d^	Butanetriol	AP	Al
HTPE system	HTPE binder	37.33	54.67	7.33	0.67		
	HTPE/AP mixture	6.72	9.84	1.32	0.12	82.00	
	HTPE propellant	5.60	8.20	1.10	0.10	68.00	17.00

^a^ dioctyl adipate; ^b^ toluene diisocyanate; ^c^ bis (2,2-dinitropropyl) formal and bis (2,2-dinitropropyl) formal acetal mixture; ^d^ isophorone diisocyanate.

**Table 2:** Propellant sampling temperatures for various degrees of thermal damage treatment.

System	Sample	Experiment Number	Sampling Temperature
AP	AP	1#	original
		2#	160 °C
		3#	180 °C
		4#	220 °C
HTPB system	HTPB binder	5#	original
		6#	160 °C
		7#	180 °C
		8#	220 °C
	HTPB/AP mixture	9#	original
		10#	160 °C
		11#	180 °C
		12#	220 °C
	HTPB/AP/Al propellant	13#	original
		14#	160 °C
		15#	180 °C
		16#	220 °C
HTPE system	HTPE binder	17#	original
		18#	160 °C
	HTPE/AP mixture	19#	original
		20#	160 °C
		21#	180 °C
		22#	220 °C
	HTPE/AP/Al propellant	23#	original
		24#	160 °C
		25#	180 °C
		26#	220 °C

**Table 3:** Flammability indices of the HTPB/AP mixture and HTPB/AP/Al propellant at various sampling temperatures.

Propellant Samples	Experiment Number	${\left(\frac{dW}{dt}\right)}_{max}$ (%/°C)	${\left(\frac{dW}{dt}\right)}_{mean}$ (%/°C)	*T_i_* (°C)	*T_h_* (°C)	*S* × 10^−8^
HTPB/AP mixture	9#	1.88	0.92	353.65	413.45	3.34
	10#	1.55	0.81	333.16	407.96	2.77
	11#	1.77	0.96	352.34	415.34	3.30
	12#	2.28	1.17	353.77	406.57	4.24
HTPB/AP/Al propellant	13#	1.83	0.84	339.22	391.82	3.41
	14#	1.19	0.66	326.11	401.91	1.84
	15#	1.42	0.71	330.85	399.05	2.31
	16#	1.45	0.66	332.83	400.23	2.42

**Table 4:** Flammability indices of the HTPE/AP mixture and HTPE/AP/Al propellant at various temperatures.

Propellant Samples	Experiment Number	${\left(\frac{dW}{dt}\right)}_{max}$ (%/°C)	${\left(\frac{dW}{dt}\right)}_{mean}$ (%/°C)	*T_i_* (°C)	*T_h_* (°C)	*S* × 10^−8^
HTPE/AP mixture	19#	2.00	0.97	353.35	411.35	3.78
	20#	1.92	0.99	342.99	399.19	4.05
	21#	1.65	0.87	337.85	408.45	3.10
	22#	1.43	0.87	333.92	405.72	2.78
HTPE/AP/Al propellant	23#	1.83	0.84	339.42	392.22	3.40
	24#	1.77	0.85	335.23	391.03	3.42
	25#	1.50	0.72	333.21	400.81	2.43
	26#	1.25	0.65	308.27	390.87	2.19

## Data Availability

The raw/processed data required to reproduce these findings cannot be shared at this time as the data also forms part of an ongoing study.

## References

[1] Zhang L.-K., Zheng X.-Y. (2018). Experimental study on thermal decomposition kinetics of natural ageing AP/HTPB base bleed composite propellant. Def. Technol..

[2] Wang Z.-J., Qiang H.-F. (2022). Mechanical properties of thermal aged HTPB composite solid propellant under confining pressure. Def. Technol..

[3] Ye Q., Yu Y.-G. (2018). Numerical simulation of cook-off characteristics for AP/HTPB. Def. Technol..

[4] Wang Z.-J., Qiang H.-F., Wang G., Geng B. (2018). Strength criterion of composite solid propellants under dynamic loading. Def. Technol..

[5] Ji Y., Cao L., Li Z., Chen G., Cao P., Liu T. (2023). Numerical Conversion Method for the Dynamic Storage Modulus and Relaxation Modulus of Hydroxy-Terminated Polybutadiene (HTPB) Propellants. Polymers.

[6] Zhang Y., Tian Y., Zhang Y., Fu X., Li H., Lu Z., Zhang T., Hu Y. (2022). Improvement in Migration Resistance of Hydroxyl-Terminated Polybutadiene (HTPB) Liners by Using Graphene Barriers. Polymers.

[7] Shi L., Fu X., Li Y., Wu S., Meng S., Wang J. (2022). Molecular Dynamic Simulations and Experiments Study on the Mechanical Properties of HTPE Binders. Polymers.

[8] Weigand A., Unterhuber G., Kupzik K., Eich T., Bucher B. (2010). Solid Propellant Rocket Motor Insensitive Munitions, Testing and Simulation.

[9] Ye Q., Yu Y., Li W. (2020). Study on cook-off behavior of HTPE propellant in solid rocket motor. Appl. Therm. Eng..

[10] SHo Y., Ferschl T., Foureur J. (1993). Correlation of Cook-Off Behaviour of Rocket Propellants with Thermal Mechanical and Thermochemical Properties.

[11] Wu X., Li J., Ren H., Jiao Q. (2022). Comparative Study on Thermal Response Mechanism of Two Binders during Slow Cook-Off. Polymers.

[12] Essel J.T., Nelson A.P., Smilowitz L.B., Henson B.F., Merriman L.R., Turnbaugh D., Gray C., Shermer K.B. (2020). Investigating the effect of chemical ingredient modifications on the slow cook-off violence of ammonium perchlorate solid propellants on the laboratory scale. J. Energetic Mater..

[13] Yan Q.-L., Zhao F.-Q., Kuo K.K., Zhang X.-H., Zeman S., DeLuca L.T. (2016). Catalytic effects of nano additives on decomposition and combustion of RDX-, HMX-, and AP-based energetic compositions. Prog. Energy Combust. Sci..

[14] Trache D., Maggi F., Palmucci I., DeLuca L.T. (2018). Thermal behavior and decomposition kinetics of composite solid propellants in the presence of amide burning rate suppressants. J. Therm. Anal. Calorim..

[15] Wu W., Jin P., Zhao S., Luo Y. (2022). Mechanism of AP effect on slow cook-off response of HTPE propellant. Thermochim. Acta.

[16] Wu W., Zhang X., Jin P., Zhao S., Luo Y. (2022). Mechanism of PSAN effect on slow cook-off response of HTPE propellant. J. Energetic Mater..

[17] Luo Y.-J., Mao K.-Z., Xia M. (2015). Effect of Hydroxyl-Terminated Random Copolyether (PET) and Hydroxyl-Terminated Polybutadiene (HTPB) on Thermal Decomposition Characteristics of Ammonium Perchlorate. J. Res. Update Polym. Sci..

[18] Mallick L., Kumar S., Chowdhury A. (2015). Thermal decomposition of ammonium perchlorate—A TGA–FTIR–MS study: Part I. Thermochim. Acta.

[19] Zhu Y.-L., Huang H., Ren H., Jiao Q.-J. (2014). Kinetics of Thermal Decomposition of Ammonium Perchlorate by TG/DSC-MS-FTIR. J. Energetic Mater..

[20] Kohga M., Togashi R. (2021). Mechanical Properties and Thermal Decomposition Behaviors of Hydroxyl-Terminated Polybutadiene/Glycerol Propoxylate Blend and Its Application to Ammonium Nitrate-Based Propellants. Propellants Explos. Pyrotech..

[21] Rocco J.A.F.F., Lima J.E.S., Frutuoso A.G., Iha K., Ionashiro M., Matos J.R., Suárez-Iha M.E.V. (2004). TG studies of a composite solid rocket propellant based on HTPB-binder. J. Therm. Anal. Calorim..

[22] Tingfa D., Junfeng L. (1991). Estimation of major volatile products from the first stage of the thermal decomposition of hydroxy-terminated polybutadiene binder. Thermochim. Acta.

[23] Wibowo H.B., Sitompul H.R.D., Budi R.S., Hartaya K., Abdillah L.H., Ardianingsih R., Wibowo R.S.M. (2022). Hexogen Coating Kinetics with Polyurethane-Based Hydroxyl-Terminated Polybutadiene (HTPB) Using Infrared Spectroscopy. Polymers.

[24] Yuan S., Zhang B., Wen X., Chen K., Jiang S., Luo Y. (2022). Investigation on mechanical and thermal properties of HTPE/PCL propellant for wide temperature range use. J. Therm. Anal. Calorim..

[25] Wang Y.-H., Liu L.-L., Xiao L.-Y., Wang Z.-X. (2015). Thermal decomposition of HTPB/AP and HTPB/HMX mixtures with low content of oxidizer. J. Therm. Anal. Calorim..

[26] Padwal M.B., Varma M. (2018). Thermal decomposition and combustion characteristics of HTPB-coarse AP composite solid propellants catalyzed with Fe_2_O_3_. Combust. Sci. Technol..

[27] Wen X., Chen K., Sang C., Yuan S., Luo Y. (2020). Applying modified hyperbranched polyester in hydroxyl-terminated polyether/ammonium perchlorate/aluminium/cyclotrimethylenetrinitramine (HTPE/AP/Al/RDX) composite solid propellant. Polym. Int..

[28] Kim K.-H., Kim C.-K., Yoo J.-C., Yoh J.J. (2011). Test-Based Thermal Decomposition Simulation of AP/HTPB and AP/HTPE Propellants. J. Propuls. Power.

[29] Zhang H., Nie J., Wang L., Wang D., Hu F., Guo X. (2022). Effect of preignition on slow cook-off response characteristics of composite propellant. Explos. Shock. Waves.

[30] Gołofit T., Ganczyk-Specjalska K., Jamroga K., Kufel L. (2018). Rheological and thermal properties of mixtures of hydroxyl-terminated polybutadiene and plasticizer (Rapid communication). Polimery.

[31] Zhang H.-J., Nie J.-X., Jiao G.-L., Xu X., Guo X.-Y., Yan S., Jiao Q.-J. (2022). Effect of the microporous structure of ammonium perchlorate on thermal behaviour and combustion characteristics. Def. Technol..

[32] Qi N. (2001). Thermogravimetric analysis on the combustion characteristics of brown coal blends. Combust. Sci. Technol..

[33] Li X.-G., Ma B.-G., Xu L., Hu Z.-W., Wang X.-G. (2006). Thermogravimetric analysis of the co-combustion of the blends with high ash coal and waste tyres. Thermochim. Acta.

[34] Yu L.J., Wang S., Jiang X.M., Wang N., Zhang C.Q. (2008). Thermal analysis studies on combustion characteristics of seaweed. J. Therm. Anal. Calorim..

[35] Xie Z., Ma X. (2013). The thermal behaviour of the co-combustion between paper sludge and rice straw. Bioresour. Technol..

